# Brain functional networks in syndromic and non-syndromic autism: a graph theoretical study of EEG connectivity

**DOI:** 10.1186/1741-7015-11-54

**Published:** 2013-02-27

**Authors:** Jurriaan M Peters, Maxime Taquet, Clemente Vega, Shafali S Jeste, Iván Sánchez Fernández, Jacqueline Tan, Charles A Nelson, Mustafa Sahin, Simon K Warfield

**Affiliations:** 1 Computational Radiology Laboratory, Department of Radiology, Boston Children's Hospital, 300 Longwood Ave-Main 2, Boston, MA 02115, USA; 2 Division of Epilepsy and Clinical Neurophysiology, Department of Neurology, Boston Children's Hospital, 300 Longwood Ave-Fegan 9, Boston, MA 02115, USA; 3 ICTEAM Institute, Université catholique de Louvain, Place du Levant 2 bte L5.04.04, 1348 Louvain-La-Neuve, Belgium; 4 Center for Autism Research and Treatment, Semel Institute 68-237, University of California, 760 Westwood Plaza, Los Angeles, CA 90095, USA; 5 Department of Child Neurology, Hospital Sant Joan de Déu, Universidad de Barcelona, Passeig Sant Joan de Déu, Esplugues de Llobregat, 08950, Barcelona, Spain; 6 VU University Medical Center, de Boelelaan 1117, 1081 HV Amsterdam, the Netherlands; 7 Laboratories of Cognitive Neuroscience, Department of Developmental Medicine, Boston Children's Hospital, 1 Autumn Street, Boston, MA 02215, USA

**Keywords:** Graph theory, Functional connectivity, Electroencephalogram, Tuberous sclerosis complex, Autism spectrum disorders

## Abstract

**Background:**

Graph theory has been recently introduced to characterize complex brain networks, making it highly suitable to investigate altered connectivity in neurologic disorders. A current model proposes autism spectrum disorder (ASD) as a developmental disconnection syndrome, supported by converging evidence in both non-syndromic and syndromic ASD. However, the effects of abnormal connectivity on network properties have not been well studied, particularly in syndromic ASD. To close this gap, brain functional networks of electroencephalographic (EEG) connectivity were studied through graph measures in patients with Tuberous Sclerosis Complex (TSC), a disorder with a high prevalence of ASD, as well as in patients with non-syndromic ASD.

**Methods:**

EEG data were collected from TSC patients with ASD (n = 14) and without ASD (n = 29), from patients with non-syndromic ASD (n = 16), and from controls (n = 46). First, EEG connectivity was characterized by the mean coherence, the ratio of inter- over intra-hemispheric coherence and the ratio of long- over short-range coherence. Next, graph measures of the functional networks were computed and a resilience analysis was conducted. To distinguish effects related to ASD from those related to TSC, a two-way analysis of covariance (ANCOVA) was applied, using age as a covariate.

**Results:**

Analysis of network properties revealed differences specific to TSC and ASD, and these differences were very consistent across subgroups. In TSC, both with and without a concurrent diagnosis of ASD, mean coherence, global efficiency, and clustering coefficient were decreased and the average path length was increased. These findings indicate an altered network topology. In ASD, both with and without a concurrent diagnosis of TSC, decreased long- over short-range coherence and markedly increased network resilience were found.

**Conclusions:**

The altered network topology in TSC represents a functional correlate of structural abnormalities and may play a role in the pathogenesis of neurological deficits. The increased resilience in ASD may reflect an excessively degenerate network with local overconnection and decreased functional specialization. This joint study of TSC and ASD networks provides a unique window to common neurobiological mechanisms in autism.

## Background

Tuberous Sclerosis Complex (TSC) is a genetic neurocutaneous disorder, with highly variable, unpredictable and potentially devastating neurological outcome [1], and approximately 40% of these patients develop autism spectrum disorders (ASD) [2]. No conventional magnetic resonance imaging (MRI) biomarker can reliably predict intractable epilepsy, cognitive impairment or autism in this population [3]. Research has conventionally focused on non-syndromic ASD, but now consensus is emerging that single gene disorders with high penetrance of ASD (for example, TSC, Fragile X syndrome, Rett syndrome) can be used to understand better the cellular and circuitry bases of ASD [4-6]. Moreover, to advance the understanding of common neurobiological mechanisms in ASD, these should be present in subjects with ASD regardless of an underlying neurogenetic abnormality. For example, using diffusion tensor imaging (DTI), we have recently demonstrated abnormalities in structural connectivity of the corpus callosum of children with TSC and a co-morbid diagnosis of ASD, adding to a growing body of evidence of callosal microstructural deficits in subjects with ASD alone [3,7-10].

Although such structural data from DTI provide insight into the architecture of interregional connections, to understand how neurophysiological function is supported by this architecture, functional networks should be analyzed as well [11]. Functional networks are implicated in cognitive functioning [12] and may form the physiological basis of information processing and mental representations [11]. They are made up by brief states of coordinated activity between physiological signals from neuronal aggregates in spatially distributed and specialized brain regions [13-16]. Functional connections form the building blocks of a functional network, and can be studied with neurophysiological techniques (for example, electroencephalography, EEG) and by neuroimaging (for example, functional MRI, fMRI).

Compared to fMRI, EEG has poor spatial resolution and is subject to volume conduction. However, it has a better signal-to-noise ratio and a significantly better temporal resolution. Moreover, EEG connectivity is directly related to neural activity, whereas fMRI is derived from the cerebral hemodynamic response to an increased metabolic demand [17], with a lag of 1 to 2 seconds from the neuronal activation. Recently, intermittent motion of the head during fMRI acquisition was shown to generate an artifactual reduction in long range connectivity and an increase in short range connectivity. This artifact may mask alterations in functional connectivity associated with autism, and complicate appropriate interpretation of functional connectivity MRI studies [18]. The best way to compensate for this artifact after the acquisition is completed remains unclear and the acquisition of MRI scans of children with autism without motion is a challenging task. On the contrary, artifact assessment is part of routine EEG interpretation by the clinical neurophysiologist, and common post-processing techniques allow for motion rejection or correction.

Thus, the main advantage of EEG is the high temporal resolution, allowing for direct characterization of higher frequency coordinated activity [19]. Recently, two data-driven analyses of EEG signals allowed for robust classification of subjects with (or at high risk for) autism and controls [20,21]. Although these EEG studies reflect functional connectivity, they do not measure the complex network properties.

To characterize these complex networks with quantitative measures, graph analysis can be applied [11]. Graph theory has been recently introduced to characterize biological systems, particularly in the brain. Graph analysis of fMRI, magnetoencephalography (MEG) and EEG signals has revealed fundamental insights into the large-scale functional organization of the human brain in health and disease. Using EEG and MEG, syndrome-specific patterns of abnormal functional networks have been described in epilepsy [22], Alzheimer's disease [14] and in adult subjects with ASD [23-25].

For ASD, graph theoretical measures of brain networks are particularly well suited as ASD is, like Alzheimer's disease, considered a disconnection syndrome [26-30]. In disconnection syndromes, functional impairment is theoretically related to the disruption or abnormal integration of spatially distributed brain regions that would normally constitute a large-scale network subserving function [11,28]. In ASD specifically, the developmental disconnection theory proposes a decreased long-range integration accompanied by increased local connectivity [7]. To synthesize the apparent inconsistencies of various long-range deficits or local surfeits in physical (DTI) and functional (fMRI) connectivity reported in ASD, a network approach may also be used [26,27,31-33].

We studied connectivity in ASD and the effects of abnormal connectivity on network properties. As the study of a homogeneous group of cooperative, high-functioning (young) adults precludes generalization of findings to the entire autism spectrum, a two-way study population was chosen: patients with and without TSC, and patients with and without ASD. To include the early developmental period of accelerated brain growth, during which autism symptoms become apparent [7] and secondary, maladaptive developmental changes have not yet occurred [34], a wide age-range was included in our study.

We hypothesized that (micro-) structural deficits in connection in TSC and ASD affect functional network properties, quantifiable by neurobiologically meaningful graph measures [35] of conventional EEG coherence. In particular, we hypothesized a widespread disconnectivity in TSC based on structural imaging findings, and decreased long-range and increased short-range connectivity in ASD, in agreement with the current model of developmental disconnection.

## Methods

### Subjects

TSC patients were identified through the Boston Children's Hospital Multidisciplinary Tuberous Sclerosis Program and were diagnosed with definite TSC based on clinical criteria described by the Tuberous Sclerosis Consensus Conference [36]. All patients with TSC were neurologically examined, and clinical data were obtained during office visits and from review of medical records. Genetic confirmatory testing included TSC1 and TSC2 gene sequencing and micro-deletion analysis at Athena Diagnostics (Worcester, MA, USA) or Boston University School of Medicine Center for Human Genetics (Boston, MA, USA). The ASD diagnoses were based on clinical assessment by a board-certified pediatric neurologist (MS and SSJ) using the Diagnostic and Statistical Manual (DSM-IV-TR), supplemented in most with the Autism Diagnostic Observation Schedule (ADOS) [37] by clinical- or research-ADOS certified specialists.

Autistic subjects without TSC (non-syndromic ASD group) were recruited from the Early Childhood Partial Hospitalization Program (ECPHP), an intensive, multidisciplinary, and highly specialized intervention program for children, two- to five-years old, with ASD via the Center for Autism Research and Treatment, Semel Institute, University of California, Los Angeles, Los Angeles, CA. ASD diagnoses were made as described for the TSC population.

Controls were selected from the general neurology clinic at Boston Children's Hospital in 2010, and were considered when they would have an EEG prompted by a single clinical event of moderate-to-low suspicion for epilepsy (for example, syncope, tics, behavioral outbursts, headache, and prominent startle). Only those subjects were included who had normal neurological development for age, a normal physical examination, a normal EEG both during wakefulness and sleep and a clinical follow-up of at least one month to confirm the trivial nature of the EEG referral. Fifteen controls had a normal imaging study, others were not imaged. Subject recruitment, data collection, retrieval and analysis were conducted with informed consent for the participation of children in the study by the parents when appropriate (for example, waived for use of retrospective EEG data), using protocols approved by the Institutional Review Boards from Boston Children's Hospital and the Semel Institute, University of California, Los Angeles.

**Figure 1:**
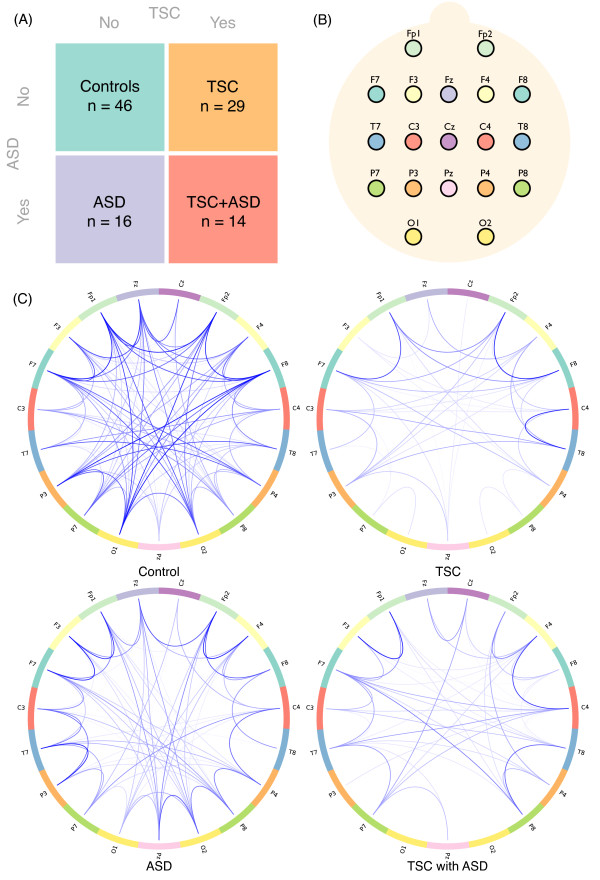
**Group structure and functional networks**. (**A**) In the two-way representation of our population, group status is defined by the subject being diagnosed with Tuberous Sclerosis Complex (TSC) or not and with Autism Spectrum Disorder (ASD) or not. This structure allows an independent attribution of effects specific to TSC and ASD. (**B**) Electrode locations from the international 10-20 system of electrode placement are used as nodes in the network. (**C**) Illustrations of the functional networks of a control subject, a TSC patient, a non-syndromic autistic patient, and a TSC patient diagnosed with autism. Colors on the connection terminations correspond to the colors in (B).

The study populations are represented in (Figure 1 A).

Previous literature has implicated an important role of the corpus callosum in ASD [8-10]. For illustrative purposes, and to ensure our functional connectivity measure was representative of callosal integrity, the effects of an absent or severely malformed corpus callosum (ACC) on coherence were assessed. These subjects were retrieved through a search of radiological reports and confirmed by review of the images and electronic medical records.

### EEG recording and artifact rejection

In Boston, through review of electronic medical records, digital EEG data were identified and retrieved from the archives. Both routine electroencephalographic data and inpatient data from long-term monitoring with video-EEG were used, utilizing the 10-20 International System of electrode placement (Figure 1 B). If multiple recordings were present, a single record was chosen based on proximity to the acquisition of imaging data, for future correlation of imaging and EEG findings. If data were of insufficient quality, the next closest EEG was chosen. EEGs were recorded on Biologic recording systems, 256 to 512 Hz sampling rate, 1 to 100 Hz bandpass, or on a Natus Neuroworks^® ^EEG system, 200 Hz sampling rate, 0.1 to 100 Hz bandpass. Data in Los Angeles were collected using a 128 Hydrocel Geodesic Sensor Net System (EGI^®^, Inc). Data were collected and recorded using NetAmps Amplifiers and NetStation software, sampled at 250 Hz, and digitized with a National Instruments Board (12 bit).

All raw data were imported, pruned, notch filtered at 60 Hz and, if necessary, spatially down-sampled to the standard clinical 19 electrodes (Figure 1B). An average reference was created using the BESA^® ^Research 3.5 software package. Next, data were imported into EEGlab for band-pass filtering (FIR filter, 1-70 Hz), rejection of artifact-ridden epochs and selection of awake task-free data, with a minimum of two minutes. Epochs with evidence of muscle artifact were, where possible, rejected. Independent Component Analysis (ICA) was used for semi-automated artifact rejection of eye blinks and lateral eye movements, according to previously described methods [38]. The average reference was used for calculation of connectivity.

After artifact removal, for each subject several segments of continuous EEG signal were available for analysis. While these segments varied in their number and length, no group difference was observed in the mean, minimum and maximum length of these segments (*P *> 0.15 for both TSC and ASD conditions). The total duration of EEG data analyzed was higher in TSC subjects (*P *< 0.01) with an average time of 646 seconds compared to 439 seconds for non-TSC subjects. No difference related to ASD was observed in the total duration of EEG data analyzed. This difference in total length was accounted for in our definition of the connectivity measure.

### Connectivity measure

The connections between brain regions that make up functional networks are measures of linear or non-linear statistical interdependence between two time-series [39,40]. Coherence is a measure of the stability of phase correlation over time and is sensitive to both changes in power and phase relationships, although the former is typically negligible [17]. High coherence values between two signals are taken as a measure of strong connectivity between the responsible brain regions [41,42]. Advantages of this measure include ample experience across the literature, the intuitive intelligibility by clinician-scientists and the description of statistically consistent, recurrent connections over a longer period of time. Drawbacks include the neglect of shorter interactions in the time domain and of non-linear relations, the assumption of stationarity of the signal, and sensitivity to volume conductance through skull, scalp and cerebrospinal fluid [14,17,42].

Combining the data from the different segments of continuous EEG recording by concatenation would span transitions which translate into artifactual high frequency content of the power spectrum. Thus, coherence was calculated for each segment individually. Our connectivity measure was obtained by computing the average of these coherences weighted by segment length. This method weighs longer segments more, and gives negligible weight to short segments. Specifically, let *S_i_(t) *be the signal in the *i*-th segment, *L_i _*be its length, *N *be the number of segments, Coh(*S,f*) be the coherence of signal *S *at frequency *f *and ϕ be the frequency band of interest, the connectivity measure read:

$$C=\underset{\phi }{\int }\frac{\overset{N}{\underset{i=1}{\sum }}L_{i}\text{Coh}\left(S_{i},f\right)}{\overset{N}{\underset{i=1}{\sum }}L_{i}}df.$$

### Group comparison of coherences

To illustrate the validity of pre-processing methods and coherence calculations, data were analyzed on a sample of 16 patients with an ACC. The corpus callosum is the largest inter-hemispheric white matter pathway, critical to direct long-range information transfer between homotopic cortical regions and is implicated in autism [3,8-10]. We calculated the ratio of the mean coherence of all corresponding inter-hemispheric electrode pairs over all non-midline intrahemispheric electrode pairs. As anticipated [43,44], decreased inter-hemispheric coherence was found in the group with an abnormal or ACC (one-tailed two-sample t-test: *P *< .006 in all three bands).

For comparison of long- versus short-range coherences, neighboring electrode pairs were ignored because of excessive volume conductance [42,45]. Short-distance mean coherence was calculated from all intra- and inter-hemispheric electrode pairs not immediately adjacent, that is, with a Euclidean distance of two. Long-distance pairs were defined as a Euclidean distance of three or more on the grid (Figure 1 B), that is, 75% or more of the maximum distance between aligned electrodes.

For analysis of TSC and ASD populations, we controlled for baseline coherences and volume conduction by comparison to healthy subjects and for maturational changes by including age as a covariate into the regression model.

The theta band (4 to 8 Hz) and the lower- and upper alpha bands (8 to 10 and 10 to 12 Hz, respectively) were chosen on the basis of previous findings in disconnection syndromes (for example, Alzheimer's disease [14] and autism (for example, [45,46])), the higher power and signal-to-noise ratio in these bands and the increased susceptibility of beta- and gamma-bands to contamination by muscle artifact in routine clinical EEG. This also allowed for limiting the number of statistical analyses.

In addition, graph analysis allows the avoidance of the multiple comparisons typically needed for group analyses at the connection level (19 electrodes have 171 possible connections in each subject). The actual correction for the comparisons of the few graph measures used would require knowing the correlation between these measures.

### Graph analysis

Mathematically, networks are represented by graphs, which consist of nodes connected by edges. A graph based on a connection measure without directionality (for example, coherence) is referred to as undirected. Graphs can also be weighted or unweighted. In an unweighted graph, edges represent the presence or absence of a connection between two nodes regardless of its strength. By contrast, weighted graphs also encode the strength of the connections within the edges. For this study, an undirected weighted graph was built using the 19 electrodes as nodes and inter-electrode coherence values as edges (Figure 1 B-C). Edge strength can be mapped to a functional distance by applying a function *f *to it [35]. We choose *f *to be the negative logarithm as it will associate a functional distance of 0 to time series in perfect synchronization (coherence equal to 1) and an infinite functional distance to incoherent time series (coherence equal to 0). Functional distances then allow the definition of functional path lengths being the sum of the functional distances along a particular path [35].

Graphs can be characterized by various global measures. It is not yet established which measures are most appropriate for the analysis of brain networks [11]. Three important ones are the characteristic path length, clustering coefficient, and global efficiency [14,35]. The characteristic path length is the average length of the shortest paths that must be traversed to go from one node to another. The clustering coefficient indicates the likelihood that two nodes strongly connected to a third node are also strongly connected to each other, forming a strongly connected triangular cluster [11]. As such, the clustering coefficient is a measure of the network segregation. The global efficiency is the average of the inverse path lengths. As a result, the global efficiency is primarily driven by shorter paths (stronger connections) while characteristic path length is primarily driven by longer paths (weaker connections). In particular, the characteristic path length of a disconnected network is infinite while its global efficiency is finite. Both characteristic path lengths and global efficiency are measures of network integration. A high clustering coefficient and a low average path length form a network with 'small-world' characteristics. Small-world architecture suggests a network with connections that are neither regular nor random, and is found ubiquitously in natural and technological systems [11,35].

Additional file 1 provides an accessible introduction to graph theory and our network measures used, as well as examples of brain and airline networks.

Another interesting property of networks is its resilience to the removal of random or highly connected nodes, known as Random Failure and Targeted Attack, respectively [43,47]. In technological networks, the resilience is typically enforced by structurally replicating the nodes, inducing a redundancy in the network. In biological systems (and in the brain in particular), nodes typically cannot be replicated and resilience may indicate that structurally different components can perform similar functions, known as functional degeneracy. Thus, while a resilient functional network may reflect the ability to preserve system function in neuropathological conditions [14,43,47], an excess of degeneracy indicates a decreased functional specialization [48-50]. To measure resilience, attacks and failures are simulated by removing nodes and their connections from the graph. The global efficiency is computed for the resulting damaged network and compared to its initial value. Global efficiency is chosen to investigate resilience, as suggested in [35].

Additional file 2 contains an entry-level description of network resilience, and the main methods of assessing resilience through the two modes of network attack. Again examples are provided for airline networks and brain networks.

All graph measures were computed using the NetworkX toolbox in Python [51] except for the global efficiency which was developed in-house.

### Statistical analysis

To distinguish the influence of ASD from that of TSC, a two-way ANCOVA was applied. This statistical model can assess effects specific to TSC and specific to ASD, and allows the inclusion of age as a covariate. However, it cannot account for differences in the group that are not additive. For example, if some measure is larger in both the ASD and TSC group but the effects do not add up in the TSC with ASD subgroup, then the group differences may not be shown by the model. Conversely, it cannot account for situations in which a group difference is attributable to a single subgroup.

Our unique two-way study population structure is somewhat akin to a repeated analysis, since, for each hypothesis tested, we have two subgroups to study. If the hypothesis is consistent for both subgroups (that is, both ASD with and without TSC, or both TSC with and without ASD), the finding is more intrinsic to ASD (or TSC).

The subject's age was used as a covariate, given the maturational changes in both EEG coherence [52,53] and in graph measures of brain networks [54,55]. The corresponding generalized linear model for each of the measures y is as follows:

$$y=\bar{y}+\beta_{ASD}ASD+\beta_{TSC}TSC+\beta_{age}age$$

where $\bar{y}$ is the baseline value, ASD and TSC are binary group variables indicating the presence or absence of ASD and TSC, respectively, and age is the subject's age in years. The two-way ANCOVA then allows us to assess whether $\beta_{ASD}$ and/or $\beta_{TSC}$ is significantly different from zero, indicating the influence of ASD and/or TSC on the measured properties.

Statistical and graph analyses were done with in-house developed software and standard issue toolbox on a MatLab platform (2009a, MatLab Inc., Natick, MA).

## Results

All results of statistical tests are displayed in Table 1.

**Table 1:** *P*-values of the differences associated with autism spectrum disorder (ASD) and tuberous sclerosis complex (TSC).

		ASD			TSC	
	
Property	Theta	Lower Alpha	Upper Alpha	Theta	Lower Alpha	Upper Alpha
Mean Coherence	0.68	0.50	0.10	0.31	0.0044(**)	0.089
Inter-Intra ratio	0.022(*)	0.26	0.47	0.14	0.78	0.58
Long-Short ratio	0.0004(***)	0.00012(***)	0.00033(***)	0.083	0.082	0.67
Clustering Coefficient	0.8	0.66	0.6	0.23	0.001(**)	0.016(*)
Average Path Length	0.5	0.57	0.085	0.33	0.0076(**)	0.17
Global Efficiency	0.71	0.31	0.2	0.37	0.0087(**)	0.11
Resilience:						
1 node removed	0.0019(**)	0.0049(**)	0.001(**)	0.097	0.74	0.39
2 nodes removed	0.0073(**)	0.06	0.0049(**)	0.19	0.53	0.45
3 nodes removed	0.003(**)	0.016(*)	0.12	0.11	0.58	0.92
4 nodes removed	0.0013(**)	0.018(*)	0.089	0.085	0.60	0.86
5 nodes removed	0.0008(***)	0.042(*)	0.094	0.10	0.74	0.88

Numbers in bold indicate significant differences between groups, asterisks are defined as follows * <0.05, ** <0.01, *** <0.001.

### Demographic data

A total of 43 subjects with TSC were included (27 male, mean age 6.9 years, range 0.7 to 25.6) and 46 age-matched control subjects (19 male, mean age 7.1 years, range 0.08 to 17.4). Fourteen TSC subjects were diagnosed with ASD (9 male, mean age 9.3 years, range 1.0 to 25.6) and 29 were not (17 male, mean age 6.0 years, range 0.7 to 23.4). Sixteen subjects with non-syndromic autism were included (12 male, mean age 4.1 years, range 2.2 to 5.5). Using Fisher's exact test (binary variables) and student t-test (continuous variables), no group differences were found in gender and age between all TSC subjects and controls (*P *= 0.45 and 0.06, respectively). Age of TSC subjects with and without autism did not differ from controls (*P *= 0.48 and 0.16, respectively). No age difference was found between all ASD subjects and controls (*P *= 0.29), but there was a slight male predominance (*P *= 0.02). Non-syndromic ASD subjects were younger than controls (mean 4.1 years +/- 1.1 versus 7.9 years +/- 5.6). Age differences were controlled for all four groups in all subsequent analyses, through the incorporation of age as a covariate into the ANCOVA model.

ASD was not associated with TSC1 or TSC2 mutations (*P *= 1.0). In all patients, there was no association between significant cognitive impairment (clinical assessment or, if available, full scale intelligence quotient < 70) and ASD (*P *= 0.15). There was no difference in the prevalence of significant cognitive impairment between patients with ASD alone and patients with ASD and TSC (*P *= 1.0). In TSC patients, there was no association between ASD and epilepsy, or ASD and infantile spasms (*P *= 1.0 and 0.19, respectively), perhaps reflecting an inclusion bias of those patients who underwent EEG recordings.

### Coherence measures

The age-related increase in mean coherence (data not shown) results from developmental changes in brain maturation [52,53]. On a network level, it represents increasing integration and decreasing segregation of structural and functional network hubs found by DTI and fMRI studies [54-56].

**Figure 2:**
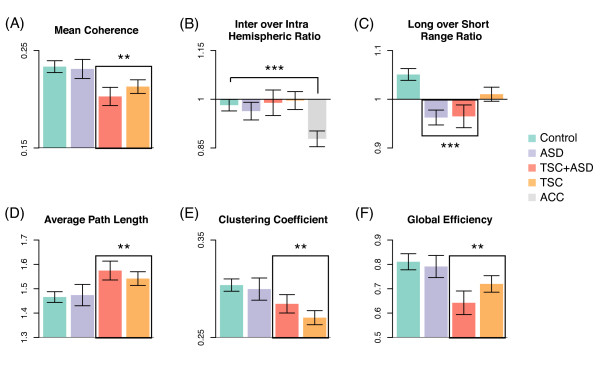
**Connectivity by measures of conventional coherence and network topology**. (A-C) Conventional coherence measures: (**A**) Mean coherence over all pairs of electrodes shows a significantly global under-connectivity for the Tuberous Sclerosis Complex (TSC) group regardless of the presence of Autism Spectrum Disorder (ASD). (**B**) Inter- versus intra-hemispheric coherence ratio. The significantly smaller value for patients with an absent corpus callosum (ACC) illustrates the validity of coherence as a connectivity measure. (**C**) Long- over short-range connectivity ratio is significantly smaller in the ASD group. As the mean coherence in (A) is not altered, this indicates a short-range overconnectivity and long-range underconnectivity in patients with ASD, evident in both subgroups (in ASD related to TSC and in ASD alone). (D-F) Network topology measures: TSC is characterized by a higher average path length (**D**) and lower clustering coefficient (**E**) and global efficiency (**F**). This departure from the small-world network topology results in a less functionally integrated and segregated network. No ASD group effect was found for these network topology measures. Only the lower alpha band is shown, see Table 1 for details. All measures are corrected for age. * *P *< 0.05, ** *P *< 0.01, *** *P *< 0.001.

In the TSC group, mean coherence between all electrode pairs was significantly decreased in the lower alpha band. This decrease indicates a significant global underconnectivity specific to TSC, corrected for age and regardless of the presence of ASD. In the ASD group, no difference in mean coherence was observed (Figure 2 A). However, note that the mean coherence does reflect the distribution of long- and short-range connections (Figure 2 C).

For TSC, despite our prior report on microstructural deficits of the corpus callosum [3], there was no difference in the ratio of inter-hemispheric over intra-hemispheric coherence (Figure 2 B).

For ASD, with the exception of a group effect in the theta band, this ratio was unchanged as well. To illustrate coherence as a measure of connectivity, this ratio indeed showed a reduction of inter-hemispheric connections in the group with an ACC [43,44].

In the TSC group, the ratio of long- over short-distance coherence trended lower but did not reach significance (Figure 2 C).

In the ASD group, this ratio was significantly and consistently decreased over all examined frequency bands. As the mean coherence (Figure 2 A) was not altered, the decreased ratio indicates a local overconnectivity accompanied by a proportional long-range underconnectivity in patients with ASD. This pattern was evident in both subgroups, that is, in both ASD with and without TSC.

### Graph measures

In patients with TSC, we found both a longer average path length (weak, long connections are weaker) and a decreased global efficiency (strong, short connections are weaker), indicating less integration through both short and long network paths (Figure 2 D-F). The clustering coefficient was decreased, indicating a decreased local connectedness in the graph. Together, the increased path length and the decreased clustering coefficient represent a network that departs from small-world topology in TSC, independent of a co-morbid diagnosis of ASD.

**Figure 3:**
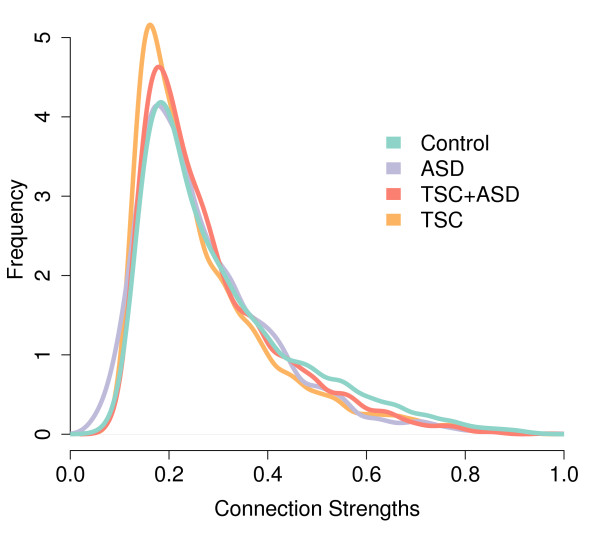
**Distribution of all connection strengths (coherences between electrode pairs)**. In Tuberous Sclerosis Complex (TSC), the distribution is left-skewed from a relative increase of lower coherence values. In Autism Spectrum Disorder (ASD), the distribution of connection strengths is similar to controls. This indicates that the alteration of the functional network in ASD stems from the allocation of similar connection strengths in a different electrode pairing scheme. Only the lower alpha band is shown.

In patients with ASD, regardless of the presence of TSC, no significant group difference was found for the three topological measures. Since in ASD the ratio of long- over short-range connectivity is significantly lower, the absence of topological differences in this population suggests that functional networks are altered while maintaining an unaltered distribution of connection strengths (Figure 3).

### Resilience measures

For TSC, there was no group effect for either the targeted attack or the random failure in all three spectral bands. The decreased mean coherence in TSC (Figure 2 A) does not affect resilience measures, as these measures reflect a percentage change relative to the baseline global efficiency of a network.

**Figure 4:**
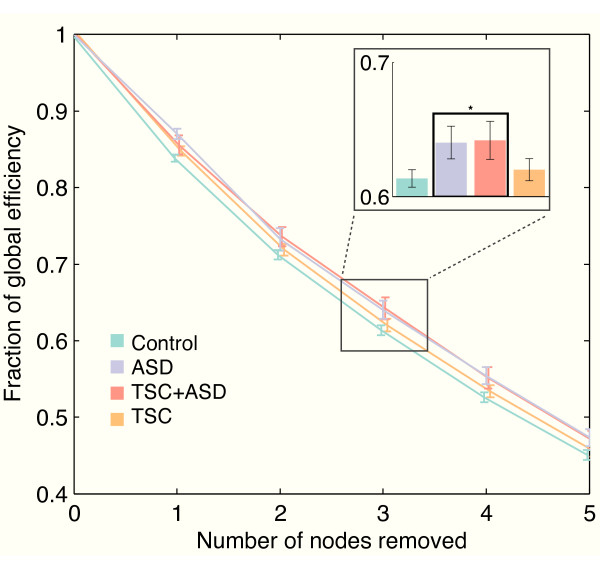
**Resilience to targeted attacks**. Autism Spectrum Disorder (ASD) is characterized by significantly higher network resilience to targeted attacks, indicating a higher degree of functional degeneracy (see discussion for details). This difference is consistent in both ASD subgroups (with and without TSC). Only the lower alpha band is shown. A sample bar plot of the global efficiency of the network after removal of three nodes is inserted (top right).

For ASD, with the targeted attack, a significantly decreased decline of the global efficiency was found. This group effect was not present with the random failure. In ASD, regardless of the presence of TSC, this significantly increased resilience to targeted attack was found in all frequency bands (Figure 4). As increased resilience could be related to an altered organization of hubs (highly connected nodes) in the network, we calculated the connection degree of the three highest connected nodes, compared to the sum of the degree of all nodes. In ASD, this normalized degree was significantly decreased across all bands (*P *< 0.005 for the first hub; *P *< 0.001 for second hub except in the upper alpha band; *P *< 0.01 for the third hub except in the upper alpha band).

### Subgroup analysis

**Figure 5:**
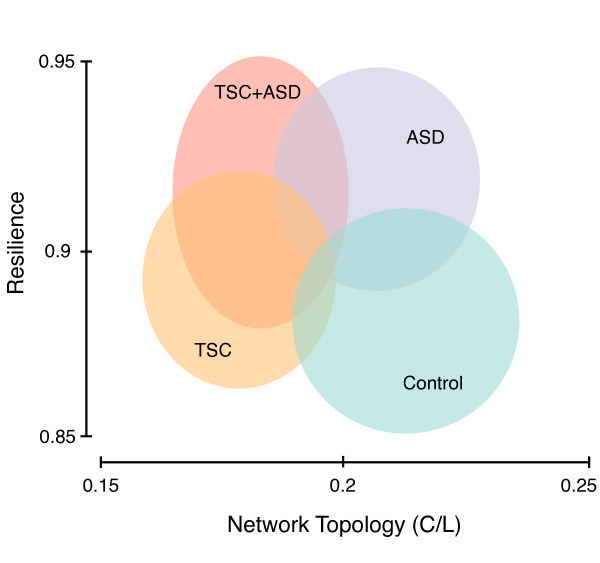
**Summary of network analysis**. In summary, functional networks in Autism Spectrum Disorder (ASD) are characterized by a high resilience to targeted attacks, and this is consistent for both ASD alone and ASD related to Tuberous Sclerosis Complex (TSC). Functional networks in the TSC group have an altered topology with a decreased clustering coefficient and increased average path length, regardless of a co-morbid diagnosis of ASD. The consistency of our findings is highlighted by the alignment of the data-cloud on the Y-axis (increased resilience for ASD) and on the X-axis (altered network topology for TSC). Groups are displayed with a span of one standard deviation.

Results in Figures 2, 3, 4 and 5 and Table 1 suggest that the observed differences are specific to a condition (TSC or ASD) rather than a subgroup. To validate this observation, we performed *post-hoc *t-tests on the differences between the subgroups. The contributions of TSC with and without ASD to the findings related to TSC (mean coherence and graph measures) were not significantly different. Similarly, the contributions of ASD with and without TSC to the findings related to ASD (long- over short-range connectivity and resilience to targeted attack) were not significantly different across all bands (except in the theta band for 1 and 2 nodes removed, *P *= 0.02 and 0.04, respectively). The difference in sub-group age between idiopathic ASD and controls did not in turn lead to the identification of a subgroup difference on these measures. There were no differences in the findings between the groups and the subgroups on these measures.

For clarity, the findings characterizing TSC by an altered network topology and ASD by an increased resilience are summarized in Figure 5.

## Discussion

We analyzed functional connectivity through EEG coherence in a large sample of children with TSC with and without ASD. Incorporating subjects with ASD alone allowed us to study connectivity abnormalities common to autism, regardless of etiology. In TSC, a pattern of global underconnectivity and altered network topology was found. ASD was characterized by a decreased long- over short-range connectivity and a markedly increased resilience to targeted attack.

### Coherence measures

In TSC, mean coherence was significantly lower, suggesting a global connection deficit. On a structural level, diffuse deficits in connectivity have also been described in TSC. First, animal models of TSC have demonstrated aberrant structural connectivity on a neural level, stemming from abnormalities in myelination, guidance and specification of the axon [57-59]. Second, in human subjects, DTI studies have revealed widespread decreased white matter microstructural integrity (for a brief overview, see [3]). This study is the first to demonstrate a functional correlate of structurally aberrant connectivity in TSC.

In ASD as well as in TSC, the ratio of inter- over intra-hemispheric connectivity did not reflect abnormalities of the corpus callosum, evident from volumetric, microstructural and functional imaging studies [3,7-9,60]. This either suggests a more subtle decrease in interhemispheric functional connectivity or more widespread distribution of altered connectivity in these disorders.

In ASD, a consistent and significantly decreased ratio of long- over short-range coherence, in the setting of an unaltered mean coherence, supports the current model of autism as a developmental disconnection syndrome. A comprehensive synthesis of prior findings in EEG and MEG coherence studies of ASD is challenging due to methodological differences [21,27]. Nonetheless, patterns of regional under-connectivity and local over-connectivity were found in several studies. Eighteen autistic adults were found to have locally increased frontal and temporal resting state theta coherence, and decreased coherence between frontal lobe and all other regions in the lower alpha band [45]. In 20 children with ASD, decreased intra-hemispheric and inter-hemispheric delta and theta coherences were reported [46]. Similar to our findings, Mathewson and colleagues found no significant difference of coherence at rest in the alpha band in adults with autism compared to controls [61]. Barttfeld *et al. *studied EEGs of 10 autistic adults with a measure of synchronization and reported a prominent deficit in long-range and an increase in short-range connectivity [25]. Our data for the first time demonstrate similar findings in both ASD associated with TSC, and in ASD alone, in support of a common mechanism.

### Graph measures

In TSC, despite extensive neurological involvement, functional connectivity has not been studied before. In ASD, the classic autistic cognitive profile of superior simple information processing and impaired higher order information processing stresses the importance of examining functional network as a whole and not only specific connections between specific regions [10]. To investigate properties of the entire functional network both in TSC and in ASD, we applied graph theoretical analysis.

The widespread deficits in local and regional connectivity in TSC are reflected both in conventional and graph measures of coherence. The aberrant network topology results in a decreased efficiency of information processing. The miswiring of axonal connections may contribute to the pathogenesis of neurological deficits in TSC [3,62,63], and our EEG study demonstrates the functional implications of these structural abnormalities on a network level.

The comparison of TSC to other disorders is complicated by the developmental rather than neurodegenerative nature of the disconnection. Using MEG, a recent study on connectivity and demyelination from multiple sclerosis reported an increased path length and clustering coefficient, suggesting a more regular network topology [64]. A similar MEG study of connectivity in Alzheimer's disease, considered a disconnection syndrome, found a decreased path length and clustering coefficient, indicating a more random network [14]. Both studies found associations between neuropsychological performance measures and graph connectivity measures, underscoring the neurobiological relevance of network analysis.

In patients with ASD, no significant differences in network topology measures were found. In the model of autism as a developmental disconnection syndrome, decreased small-worldness could be anticipated, as demonstrated in a MEG study using the synchronization likelihood in young adults with high-functioning autism [25]. Decreased small-worldness may then represent a decrease in local specialization (lower clustering coefficient) and regional integration (longer average path length) [65]. Our data may not have shown this due to the young age of the study population, where such refinement has not taken place yet [56]. Also, the absence of a higher clustering coefficient and of a longer path length for ASD despite the decrease in long-over-short range coherence demonstrates that nodes are spatially more clustered (conventional analysis) but not functionally more clustered (network analysis). This discrepancy is based on the conceptual difference between 'physical distance' and 'network distance'.

### Resilience

In the ASD group, a key finding of significantly increased resilience to targeted attack was found. Different explanations to this observation can be posited, each reflecting different aspects of proposed neurobiological mechanisms of ASD.

First, increased resilience in the networks of autistic subjects could be related to redundant connectivity patterns. An abundance of connections makes a network highly resilient to attacks. However, connections in the brain are formed at a high physical cost [11,66] and the brain constantly negotiates the trade-off between wiring costs and topological efficiency [67]. In particular, normal early developmental overconnectivity is followed by a pruning of connections in the maturing brain [55], suggesting network refinement [56].

Physiologically, in autism, the redundancy of connections could be explained by an impaired pruning of connections in the aforementioned dynamic process. The remaining overconnected network operates at different scales, from the neuronal level to the system level, and is consistent with studies of early cerebral overgrowth in autism (for a summary, see [7,26]).

Cognitively, overconnectivity can result in a poor signal-to-noise ratio where the system is flooded with noise that swamps the signal [34]. With a poor signal-to-noise ratio, the output of a network may not be sufficiently distinct to achieve the necessary information processing [30]. Thus, overconnectivity can create an abnormally undifferentiated response to any stimulus. This excess of information gets equal rather than selective attention, creating an overstimulated, inefficient and delayed processing bottleneck [34].

Second, increased resilience could imply decreased functional specialization of brain regions. In technological systems, resilience refers to redundancy, as the same function is performed by identical elements. In biological networks, however, it refers to degeneracy, as structurally different elements can perform the same function [48,50]. A degenerate system implies less specialization as the same output can be generated by different elements. Therefore, the increased resilience found in ASD could indicate an excessively degenerate system, where the removal of targeted nodes does not much affect the global properties of the network. Their presence is apparently less critical to the network, providing evidence of decreased functional specialization of these nodes. Our finding of a decreased level of connectivity of the main three hubs in the ASD population adds support to this interpretation.

In summary, the integration of primary order perceptions into higher order concepts is altered in ASD, but whether this is a top-down deficit (developmental disconnection syndrome) or due to heightened primary processing remains unclear [28]. A study using local coherence measures has argued that decreased responsiveness of autistic subjects to external stimuli may stem from a signal reduction through excess dampening [21]. Our network approach, however, suggests it rather comes from an excess of information processed in an overconnected, less specialized network [7,31].

### Future directions

Nodes should best represent brain regions with coherent patterns of extrinsic anatomical or functional connections which is problematic with EEG [35]. Specifically, node definition with only 19 electrodes is problematic as the locations do not match well-defined functional regions. Higher density EEG with 128 or 256 channels in an experimental setting can overcome this problem in part, although, inherent to the technique, only superficial aspects of the brain network can be modeled. In addition, electrode positions relative to underlying anatomical structures and functional areas are subject to variability, to changes related to growth and maturation and to methods of electrode placement used (for example, high density electrode cap or net). Thus, the level of anatomic accuracy of the current study does not allow for examining in detail the relation between functional and structural connectivity. Volume conduction will result in lattice-like graphs with highly clustered connections between neighboring electrodes, potentially confounding analysis of network properties [66]. Other measures of connectivity are less sensitive to this problem such as the phase lag index, synchronization likelihood [14,68,69], and partial directed coherence [17]. Nevertheless, it is encouraging that many 'headline' results seem to be robust to methodological details at several steps of network generation [70].

Frequency bands clearly have different associations with different aspects of cognitive activity, with different roles in pathology and with different biophysical mechanisms, reviewed in [71,72]. Also, connectivity levels between regions are different for each frequency band [73]. Some authors have proposed that long distance communication may be mainly reflected by synchronization in low frequency bands (alpha and theta range) while shorter distance local communication is supported by synchronization in beta and gamma frequency bands [74]. As a result, it is imperative that any study should try to be as complete as possible in investigating connectivity in the different bands. However, to limit the number of comparisons, but more importantly because of potential muscle artifact (interference with beta and gamma bands) and potential residual motion artifact (interference with the delta band) in the EEG data of this challenging population, our study was restricted to the theta, upper and lower alpha bands.

Bands not studied include the delta band and the beta and gamma bands. Slower brain oscillations in the delta and sub-delta range appear to have a physiological role in sensory processing and cognition, even in the absence of environmental stimulation (for example, the default mode network in resting EEG and fMRI studies) [75]. In autistic patients, Coben and colleagues found the most significant coherence changes in autistic patients in the delta and theta bands compared to controls [46]. Gamma frequency oscillatory activity has been implicated in local cognitive processes and in the development of distributed cortical networks through both resting-state and task-related neural synchrony in this band [76]. Unfortunately, while analysis of these bands would be no additional challenge to execute, with the current limitations of the clinically acquired data, the findings would not be meaningful.

Finally, the heterogeneity of the study population should be emphasized. First, the use of clinical EEG data as controls could have introduced a bias from subtle EEG abnormalities that have escaped routine interpretation by the clinical neurophysiologist, and controls were recruited from a population with neurological complaints. Second, autism is a spectrum disorder with a wide range of severity, not well reflected by a binary variable (presence or absence of ASD). In future studies continuous variables could be used, such as the calibrated severity score of the ADOS [77], or the Social Responsiveness Score (SRS), which was recently used in another cross-disorder approach of autism [78]. Third, patients with TSC have wide variability in their phenotypical presentation. We did not incorporate anti-epileptic or psychoactive medication, and epilepsy-severity variables into the model, while in TSC up to 90% experience seizures in their lifetime [1]. With this high co-occurrence of epilepsy, cognitive impairment and autism in TSC [1], differences found may represent more global neurocognitive and behavioral dysfunction in TSC [78], although in our patients we did not find an association between severe cognitive impairment and autism. While EEG segments with epileptic discharges were excluded from analysis, it remains possible that epilepsy impacted the network analysis, in particular of the TSC population. However, interictal functional networks of patients with epilepsy are characterized by increased connectivity (especially in the theta band) and topological changes including increased regularity and hub-like organization [79-82] which we did not find.

In summary, several limitations of our study including its retrospective nature, the sources of EEG data and the possibility of different cognitive states of the subjects [27] can largely be addressed by a prospective study design. The burden associated with an EEG procedure is especially prominent in the young, low-functioning autistic population and may only be justified by clinical indication [21] warranting the exploratory use of already collected data. Still, a large, prospective, multicenter endeavor for determination of advanced neuroimaging and EEG correlates of autism in TSC has been launched. In addition, graph theoretical analysis of resting state fMRI connectivity in our population could validate our findings with much higher spatial resolution.

## Conclusions

Connectivity analysis can provide fundamental insights into temporal functional coupling of spatially separate, specialized brain regions. Our EEG coherence study demonstrates decreased functional connectivity related to TSC in a global manner and to ASD in a more complex pattern.

In TSC, this study is the first to demonstrate altered functional connectivity, both through direct measurement of EEG coherence and on a network level. These results may represent a functional correlate of structural connectivity abnormalities in TSC and contribute to the neurological pathogenesis in TSC.

In ASD, a decreased long-over short-range coherence and markedly increased resilience to targeted attack renders an excessively degenerate network with local overconnection and decreased functional specialization.

## Abbreviations

ACC: absent corpus callosum; ADOS: Autism Diagnostic Observation Schedule; ANCOVA: analysis of covariance; ASD: Autism Spectrum Disorder; C: control; DSM: Diagnostic and Statistical Manual; DTI: diffusion tensor imaging; EEG: electroencephalography; (f)MRI: (functional) magnetic resonance imaging; MEG: magnetoencephalography; TSC: Tuberous Sclerosis Complex.

## Competing interests

The authors declare that they have no competing interests.

## Authors' contributions

JMP and MT conceived and designed the experimental approach, performed data collection, data analysis and prepared the manuscript. CV was critical for analysis and interpretation of phenotypical data. ISF and JT assisted with data collection and pre-processing. CN and SSJ assisted with data collection and significant edits of the manuscript. MS and SKW supervised the project, and contributed to experimental design and analysis. All authors read and approved the final version of the manuscript.

## Authors' information

JMP is a pediatric neurologist, epileptologist and clinical neurophysiologist with an interest in EEG signal processing. He aims to increase objectivity of EEG interpretation in the clinical setting, and improve availability to the non-neurophysiologist by translating the signal to colors, numbers or graphs. In the Computational Radiology Laboratory, his research focuses on establishing imaging and neurophysiological correlates of the neurological phenotype in Tuberous Sclerosis Complex (TSC), a neurocutaneous disorder strongly but unpredictably associated with epilepsy, cognitive disability and autism. MT is an electrical engineer and graduate student in neuroimaging. His work focuses on the development of novel image processing and statistical techniques to carry out population studies of the brain structure and function. In particular, he aims to better understand and analyze the brain network and its resilience. His contributions have been applied to the analysis of neurological differences in tuberous sclerosis complex and autism.

## Pre-publication history

The pre-publication history for this paper can be accessed here:

http://www.biomedcentral.com/1741-7015/11/54/prepub

## Supplementary Material

Additional file 1 **Graph Analysis: An introduction**. Description: An easily accessible introduction to graph theory and network analysis. Each measure is explained with both text and illustration, and for each an example is given of airline networks and brain networks. Click here for file

Additional file 2 **Graph Analysis: Resilience**. Description: An entry-level description of the concept of network resilience and the main methods of assessing resilience through two modes of attack of the network. Examples are provided for airline networks and brain networks. Click here for file
