# Supplementary material for: Brain functional networks in syndromic and non-syndromic autism: a graph theoretical study of EEG connectivity
Source: BMC Med. 2013 Feb 27;11:54. doi: 10.1186/1741-7015-11-54 (PMC3626634; doi:10.1186/1741-7015-11-54)
Supplement: Additional file 1 — Graph Analysis: An introduction. Description: An easily accessible introduction to graph theory and network analysis. Each measure is explained with both text and illustration, and for each an example is given of airline networks and brain networks. [file 1741-7015-11-54-S1.PDF]

Box 1 Graph Analysis: An introduction
Graphs are mathematical representations of networks in which nodes are connected by edges (see figure below). In the case of an airline network, for example, the nodes are the airports and the edges are the flights connecting them. In the case of brain networks, nodes correspond to brain regions (e.g. electrodes in EEG) and edges represent the connections between them. Graphs can be weighted or unweighted. In weighted graphs, edges are weighted by the strength of the connection. In the figure, the strength is represented by the darkness of the edges. The strength can then be used to define a network distance, which increases when the strength decreases. In the airline network, the duration of the flight can be used as a temporal distance between airports. In the brain network, edges can be weighted by the coherence between times series. A functional distance can be computed by applying a function *f* to the coherence. Once the graph is constructed, different measures can be computed to investigate different properties of the network.

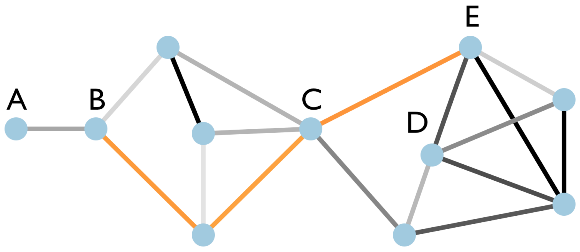

Average path length
Description: Different paths may connect one node to another (path from B to E). The *shortest path length* is the total distance of the shortest path. Averaging these shortest path lengths for all pairs of nodes yields the *average path length*. This graph measure reflects the integration of the network, i.e. how well connected distributed regions are.

Airline networks: The average path length is the average time needed to go from any airport to any other. The average path length is high if there are poorly connected airports (node A). To decrease it, airline companies would need to make sure no small airport is isolated.

Brain networks: The average path length is the average functional distance between any two brain regions. It is a measure of how rapidly information from different specialized regions can be combined.

Clustering coefficient
Description: The clustering coefficient indicates the likelihood that two nodes strongly connected to a third node are also strongly connected to each other, forming a strongly connected triangle in the graph (ref Rubinov and Sporns). In the figure, the clustering coefficient is higher in the right part of the graph than in the left part. The clustering coefficient is considered a measure of the network *segregation*. Networks that are both integrated and segregated are commonly called *small world networks*.

Airline networks: The clustering coefficient is high if regional airports (e.g. Long Beach, CA, and Monterey, CA) have direct connections to each other and not just to major hubs (e.g. New York JFK). Thanks to small-world properties, one could fly from Long Beach, CA to Bordeaux, France, with only two transfers in major hubs.

Brain networks: In the brain, a high clustering coefficient indicates the presence of local cliques forming specialized functional units. Brain networks are small-worlds in which different functional units can work independently but are connected to each other through hubs.

Global efficiency
Description: The global efficiency is another measure of network integration. It is defined as the average of the inverse path lengths. In contrast to the average path length that is very affected by long path with isolated

nodes, the global efficiency is mostly driven by short paths between hubs. The graph in the figure would have a larger efficiency if nodes C and D were directly connected.

Airline networks: The global efficiency is high when fast direct flights exist. To increase it, airline companies can introduce fast planes to connect hubs (like the Concorde that used to connect Paris, London and New-York).

Brain networks: In brain networks, a high global efficiency indicates that functional units are well integrated. It is primarily influenced by strong direct connections, which, in EEG analysis, are more physiologically meaningful than long paths between nodes.
