# Supplementary material for: Brain functional networks in syndromic and non-syndromic autism: a graph theoretical study of EEG connectivity
Source: BMC Med. 2013 Feb 27;11:54. doi: 10.1186/1741-7015-11-54 (PMC3626634; doi:10.1186/1741-7015-11-54)
Supplement: Additional file 2 — Graph Analysis: Resilience. Description: An entry-level description of the concept of network resilience and the main methods of assessing resilience through two modes of attack of the network. Examples are provided for airline networks and brain networks. [file 1741-7015-11-54-S2.PDF]

Box 2 Graph Analysis: Resilience

The resilience of a network to the removal of nodes provides insight into the presence of spare connections. Spare connections allow for alternative paths to be crossed in the network when the shortest path can no longer be taken due to missing nodes. Resilience is typically measured by simulation of node removal in the network, either as a *random failure* or a *targeted attack*. Resilience of the airline network would allow one to get to its destination even if one airport is closed due to weather conditions. In brain networks, resilience may indicate *degeneracy*, where a path through functionally different nodes yields the same output.

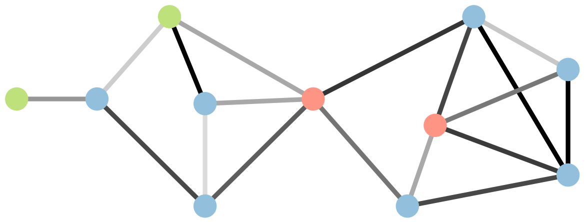

Random failure
Description: With each next random omission of nodes (green nodes in the figure), the decline in the graph performance is measured (typically global efficiency, see Box 1).

Airline networks: Due to progressively worsening international strikes of ground-workers, more and more random airports are temporarily closed, leading to inefficient travel.

Brain networks: Multifocal head trauma could be viewed as an injury to randomly distributed regions. With scattered small injuries, large neurological deficits may be limited, indicating resilience to random failure.

Targeted attacks
Description: Hubs with a high degree centrality are removed first (red nodes in the figure).

Airline networks: A global terrorist group executes a coordinated sequential bomb-attack against the control towers of major international airports. International travel and travel between major metropolitan areas become impossible.

Brain networks: A targeted attack may better mimic preferential neurodegeneration of highly connected hubs (ref Stam). Physiologically, such attacks may be more plausible, as highly connected nodes tend to be more metabolically active, rendering them more vulnerable for neurodegenerative disease (ref Alstott). Excessive resilience could indicate that removal of such active hubs is less critical to network function, suggesting decreased specialization.
